
JOURNAL INFORMATION
==============================
NLM Title Abbreviation: ScientificWorldJournal
Journal ID: ScientificWorldJournal
NLM Journal ID: 101131163
Journal ID: TSWJ

The Scientific World Journal

ISSN: 2356-6140
EISSN: 1537-744X
Publisher: Wiley

ARTICLE INFORMATION
==============================
PMCID: PMC5901287
DOI: 10.1100/tsw.2007.235
Article ID: 464603
Article version: 1
Subjects: Publisher's Note

Publication of a Special Issue “Glucocorticoids and Melanocortins: Hormones with important Effects on Immunity”

Redhead Claire
Publication date: 2007
Electronic publication date: 2007 Sep 1
Volume: 7
First page: 1465
Last page: 1465
Copyright: Copyright © 2007 Claire Redhead.
Copyright year: 2007
License: This is an open access article distributed under the Creative Commons Attribution License, which permits unrestricted use, distribution, and reproduction in any medium, provided the original work is properly cited.
License URL: https://creativecommons.org/licenses/by/3.0/

==============================
Publisher's Note
Special Issue: Glucocorticoids and Melanocortins
*TheScientificWorld,* (2007) 7, 1465
ISSN 1537-744X; DOI 10.1100/tsw.2007.235

TheScientificWorldJOURNAL
www.thescientificworld.com

### Publication of a Special Issue 'Glucocorticoids and Melanocortins: Hormones with Important Effects on Immunity'

Claire Redhead
*TheScientificWorld*

**E-mail:** credhead@thescientificworld.com

*Published September 1, 2007*

---

TheScientificWorldJOURNAL is delighted to have published the special issue 'Glucocorticoids and Melanocortins: Hormones with important effects on immunity', an important collection of peer-reviewed papers covering key topics. The special issue has been organized by Dr Stephen J. Getting (Department of Human and Health Sciences, University of Westminster, London, UK) and investigates the issue explores the role that melanocortins and glucocorticoids play in modulating the host inflammatory response.

Specific topics covered are:

- Antimicrobial Properties of á-MSH and Related Synthetic Melanocortins
- Mode of Glucocorticoid Actions in Airway Disease
- Lipoxins and Annexin-1: Resolution of Inflammation and Regulation of Phagocytosis of Apoptotic Cells
- Annexin 1 and Melanocortin Peptide Therapy for Protection Against Ischaemic-Reperfusion Damage in the Heart
- Dissociated Steroids
- Effects of Glucocorticoids on Apoptosis and Clearance of Apoptotic Cells

The Publisher wishes to express thanks to all of the authors and reviewers, and especially to acknowledge the contribution of Dr. Getting in bringing about the publication of these papers.

**This article should be cited as follows:**

Redhead, C. (2007) Publication of a special issue 'Glucocorticoids and melanocortins: hormones with important effects on immunity'. *TheScientificWorldJOURNAL* **7**, 1465. DOI 10.1100/tsw.2007.235.

©2007 with author.
